# AFM Investigation of the Influence of Steam Flow through a Conical Coil Heat Exchanger on Enzyme Properties

**DOI:** 10.3390/mi13122041

**Published:** 2022-11-22

**Authors:** Yuri D. Ivanov, Ivan D. Shumov, Vadim Y. Tatur, Anastasia A. Valueva, Andrey F. Kozlov, Irina A. Ivanova, Maria O. Ershova, Nina D. Ivanova, Igor N. Stepanov, Andrei A. Lukyanitsa, Vadim S. Ziborov

**Affiliations:** 1 Institute of Biomedical Chemistry, Pogodinskaya Str., 10 Build. 8, Moscow 119121, Russia; 2 Joint Institute for High Temperatures of the Russian Academy of Sciences, Moscow 125412, Russia; 3 Foundation of Perspective Technologies and Novations, Moscow 115682, Russia; 4 Moscow State Academy of Veterinary Medicine and Biotechnology Named after Skryabin, Moscow 109472, Russia; 5 Faculty of Computational Mathematics and Cybernetics, Moscow State University, Moscow 119991, Russia

**Keywords:** horseradish peroxidase, enzyme aggregation, atomic force microscopy, triboelectric effect, coiled heat exchanger, superheated steam

## Abstract

The present study is aimed at the revelation of subtle effects of steam flow through a conical coil heat exchanger on an enzyme, incubated near the heat exchanger, at the nanoscale. For this purpose, atomic force microscopy (AFM) has been employed. In our experiments, horseradish peroxidase (HRP) was used as a model enzyme. HRP is extensively employed as a model in food science in order to determine the influence of electromagnetic fields on enzymes. Adsorption properties of HRP on mica have been studied by AFM at the level of individual enzyme macromolecules, while the enzymatic activity of HRP has been studied by spectrophotometry. The solution of HRP was incubated either near the top or at the side of the conically wound aluminium pipe, through which steam flow passed. Our AFM data indicated an increase in the enzyme aggregation on mica after its incubation at either of the two points near the heat exchanger. At the same time, in the spectrophotometry experiments, a slight change in the shape of the curves, reflecting the HRP-catalyzed kinetics of ABTS oxidation by hydrogen peroxide, has also been observed after the incubation of the enzyme solution near the heat exchanger. These effects on the enzyme adsorption and kinetics can be explained by alterations in the enzyme hydration caused by the influence of the electromagnetic field, induced triboelectrically by the flow of steam through the heat exchanger. Our findings should thus be considered in the development of equipment involving conical heat exchangers, intended for either research or industrial use (including miniaturized bioreactors and biosensors). The increased aggregation of the HRP enzyme, observed after its incubation near the heat exchanger, should also be taken into account in analysis of possible adverse effects from steam-heated industrial equipment on the human body.

## 1. Introduction

The motion of various liquid [1,2,3,4,5,6,7,8], gaseous [9,10], and two-phase [11,12,13,14] media along solid surfaces is known to cause the so-called triboelectric effect, which consists in the generation of an electric charge. The triboelectric effect in liquid media is now actively studied, being utilized in triboelectric nanogenerators [3,4,5,12,13,15,16]. The electric charge, generated in such a way, accordingly induces electric/electromagnetic fields. In this regard, the occurrence of electromagnetic fields induced triboelectrically upon the motion of water [6,17] and non-aqueous liquids [7,8,18,19] through pipes—including coiled ones [6,7]—should be mentioned. Coiled pipes (or simply coils) find numerous applications in heat exchanging equipment [20,21,22]. These heat exchangers can be organized in the form of cylindrical [22] and conical [23,24,25] coils.

In industrial coil heaters, steam is often employed as a heat-transfer agent [26]. In this connection, one should emphasize the occurrence of significant electrostatic effects upon the motion of steam [27,28,29,30]. These effects can even cause emergency situations in industry [31]. Accordingly, further investigation of these effects is required in order to develop safety standards regulating the steam-carrying equipment operation.

Electromagnetic [32,33,34,35,36,37,38,39] and magnetic [40,41,42,43] fields are known to affect physicochemical properties of enzymes. With regard to triboelectrically induced fields, they were reported to influence adsorption properties [6,7,8] and enzymatic activity [7] of horseradish peroxidase (HRP), which is often used as a model in studying the effects of electromagnetic and magnetic fields on enzymes [6,7,8,32,33,34,36,37,38,39,40,41,42,43]. Enzyme systems play key roles in the regulation of metabolic processes in the body [44]. This is why it is quite important to study the possible influence of electromagnetic fields, induced in steam-carrying heat exchangers, on enzyme systems.

The study of peroxidases is of great interest because these enzymes are well-represented in plant and animal tissues [44] and play important functional roles in the body. In the human body, in particular, an important role of myeloperoxidase involved in atherogenesis should be mentioned [45]. HRP is a ~44 kDa heme-containing enzyme [46,47], which is widely employed as a model in food science [36,37] in order to determine the influence of electromagnetic fields on enzyme systems [36,37,38,39]. HRP finds numerous applications in biotechnology [48,49] and in miniaturized biosensor systems [50,51], and this is another reason why it is extensively studied.

In the present work, with the example of HRP, we investigated whether the motion of steam through a conical heat exchanger affects the properties of the enzyme. The solution of HRP was incubated either near the apex or at the side of the conically wound aluminium pipe, through which steam flow passed. In order to study the adsorption properties and aggregation state of HRP before and after the incubation of its solution near the heat exchanger, atomic force microscopy (AFM) was used, while the HRP enzymatic activity was studied by spectrophotometry. 

Owing to its ultra-high (0.1 nm) height resolution, AFM represents a powerful tool, which is widely employed for single-molecule investigation of enzymes [52,53,54,55,56,57,58,59,60,61,62]. In this way, AFM was employed to investigate the immobilization of ferredoxin-NADP^+^ reductase [52] and HRP [53] onto silanized mica. AFM was widely employed to reveal the aggregation state of HRP [6,7,8,32,33,34] and CYP102A1 [54] enzymes, and to study complex formation in the CYP11A1 enzyme system [55]. Berge et al. revealed a dimerization of the *Eco*KI enzyme after its binding with a DNA containing two recognition sites for the enzyme—as opposed to the case with a DNA containing one recognition site, when only a monomeric enzyme was observed [56]. By high-speed AFM, Crampton et al. visualized the interaction of *Eco*P15I with DNA, revealing two distinct mechanisms of this interaction [57]. By AFM, van Noort et al. [58] observed association, dissociation, and movement of photolyase over DNA macromolecules. Furthermore, in a number of publications, Radmacher and colleagues reported the use of an AFM-based approach for the direct observation of enzyme activity, which manifested itself in the form of height fluctuations of enzymes upon their interaction with respective substrates [59,60]. Namely, 1 nm height fluctuations of lysozyme macromolecules were revealed in the presence of an oligosaccharide substrate; moreover, such fluctuations were not observed without the substrate, or in the presence of lysozyme inhibitor chitobiose [59]. Measuring such height fluctuations allows one to directly observe single catalytic events of the enzyme; this has also been demonstrated with the example of chitosanase from *Streptomyces griseus* [60]. In [61], with the example of urease enzyme, immunoglobulin G, and microtubules, differences in height fluctuations above different macromolecules were revealed. Moreover, the use of AFM for studying lateral drift rate of urease macromolecules on silanized glass substrates was demonstrated [61]. Ivanov et al. [62] revealed that the amplitude of height fluctuations of oligomeric CYP102A1 enzyme was higher than that of monomeric CYP102A1 in the first 100 s of the enzyme functioning. After 100 s, a drop in the height fluctuation amplitude was observed, and this drop was explained by possible self-degradation of the enzyme [62]. 

**Figure 1:**
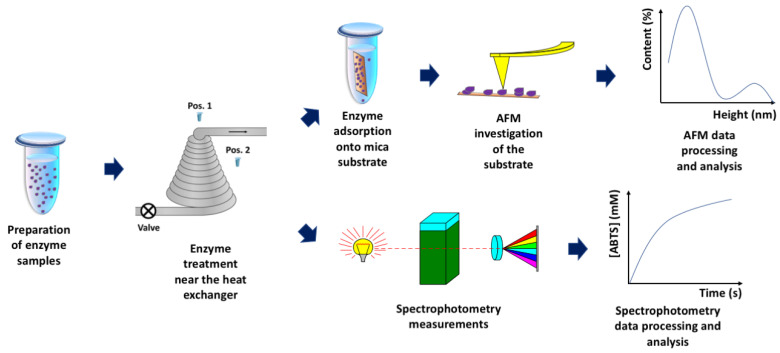
Schematic representation of general workflow of the experiments performed in order to investigate the influence of steam flow in conical heat exchanger on HRP enzyme.

The above-mentioned studies clearly demonstrate the ability of AFM to reveal even subtle effects of external factors on enzyme macromolecules [34]. Such subtle effects are often indistinguishable by macroscopic methods and can only be revealed by nanotechnology-based methods such as AFM [6,32,33,34]. This is why this method has been employed herein. This study has been aimed at the investigation of the influence of steam flow in a conical coil heat exchanger on individual HRP macromolecules incubated in its vicinity. The adsorption of HRP on mica has been investigated by AFM at the level of individual enzyme macromolecules. In parallel, spectrophotometry measurements of the HRP enzymatic activity in solution have been performed. Figure 1 displays the general workflow of the experiments performed.

By AFM, we demonstrated that the flow of superheated steam in the conical coil affects the adsorption properties of HRP macromolecules on mica. Namely, for the first time, an increased aggregation of the HRP enzyme on the mica substrate has been observed by AFM after its incubation either near the top or at the side of the conical heat exchanger. At the same time, such an incubation has been found to cause a change in the shape of the kinetic curve reflecting the HRP-catalyzed oxidation of its substrate 2,2′-azino-bis(3-ethylbenzothiazoline-6-sulfonate) (ABTS). The results obtained herein should be taken into account in the development of equipment involving conical heat exchangers, intended for either research or industrial use. Additionally, our data reported can also contribute to further analysis of possible adverse effects from steam-heated industrial equipment on human body.

## 2. Materials and Methods

### 2.1. Chemicals and Enzyme

Peroxidase from horseradish (Cat. #6782), and its substrate 2,2′-azino-bis(3-ethylbenzothiazoline-6-sulfonate) (ABTS; Cat. #A1888) were purchased from Sigma (St. Louis, MO, USA). Disodium hydrogen orthophosphate (Na_2_HPO_4_), citric acid, and hydrogen peroxide (H_2_O_2_) were all of analytical or higher purity grade, and were purchased from Reakhim (Moscow, Russia). Dulbecco’s modified phosphate buffered saline was prepared by dissolving a salt mixture, commercially available from Pierce (Waltham, MA, USA), in ultrapure water. All solutions used in our experiments were prepared using deionized ultrapure water (with 18.2 MΩ × cm resistivity), obtained with a Simplicity UV system (Millipore, Molsheim, France).

### 2.2. Experimental Setup

**Figure 2:**
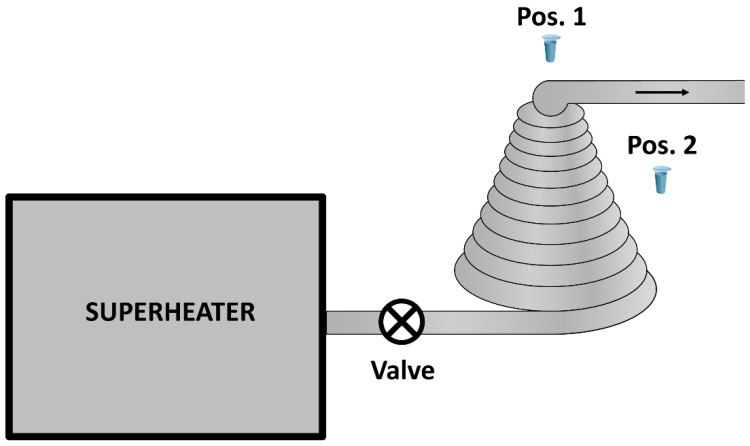
Experimental setup. Arrow indicates the direction of the steam flow. The heat exchanger was covered with a thermal shield.

In order to investigate the influence of steam flow through a conical coil heat exchanger on HRP, we used an experimental setup, which is schematically shown in Figure 2.

In the setup, superheated water steam was generated by means of a 20 L superheater operating at a pressure of 190 atm. After water in the superheater reached a temperature of 190 °C, the valve was opened, and the superheated steam passed through the conical coil, exiting through the linear output part of the coil. The temperature distribution was as follows: at the coil input, the steam temperature was 100 °C; at the cone half-height, the temperature decreased to 84 °C; at the top of the cone, the temperature was 75 °C; and at the heat exchanger output (40 cm away from the cone), the steam temperature was 70 °C. The temperature was measured with an RST RST07851PRO contact thermometer (RST, China). The steam flow time was four minutes. The coil was formed using an aluminium pipe, and had the following dimensions: the base diameter was 80 cm, the apex angle was 51°, and the height was 90 cm. The heat exchanger was covered with a thermal shield, fabricated from metallized polypropylene. The test tube with 1 mL of 10^−7^ M HRP solution in 2 mM, pH 7.4 Dulbecco’s modified phosphate buffered saline (PBSD) was placed either 2 cm above the top (Pos. 1 in Figure 1) or at the side (Pos. 2 in Figure 1) of the conical coil, and incubated there for three minutes. The control enzyme sample in the same test tube was placed 50 m away from the experimental setup.

After the incubation near the conical coil, the enzyme solution was investigated by AFM and by spectrophotometry according to the techniques described in our previous papers [6,7,8,32,33,34].

### 2.3. Atomic Force Microscopy

The AFM samples were prepared using the direct surface adsorption method developed in [63] according to the well-established technique described in detail in our previous papers [6,7,8,32,33,34]. Mica AFM substrates with adsorbed HRP were investigated with a Titanium multimode atomic force microscope (NT-MDT, Zelenograd, Russia; the microscope pertains to the equipment of “Human Proteome” Core Facility of the Institute of Biomedical Chemistry, supported by Ministry of Education and Science of Russian Federation, agreement 14.621.21.0017, unique project ID: RFMEFI62117X0017). The microscope was equipped with NSG10 cantilevers (TipsNano, Zelenograd, Russia; 47 to 150 kHz resonant frequency, 0.35 to 6.1 N/m force constant). After processing the AFM data, relative distributions of the visualized HRP particles with height (*ρ*(*h*) distributions) were calculated using the software developed at the Institute of Biomedical Chemistry in collaboration with Foundation of Perspective Technologies and Novations as described by Pleshakova et al. [64]:

*ρ*(*h*) = (*N_h_*/*N*) × 100%, (1)

where *N_h_* is the number of imaged enzyme particles of height *h*, and *N* is the total number of the imaged particles [64]. The number of frames obtained for each substrate was ≥10. For each enzyme sample studied, the AFM measurements were performed in at least three independent technical replicates. Blank experiments were performed with the use of enzyme-free buffer instead of HRP solution, and no objects with heights exceeding 0.5 nm were detected in the blank experiments.

### 2.4. Spectrophotometry

HRP activity was estimated according to the technique described in detail by Sanders et al. [65] using ABTS as the HRP substrate. The measurements were performed as described in our previous papers [6,7,8,32,33,34] in phosphate-citrate buffer with pH 5.0 [65] with an Agilent 8453 UV-visible spectrophotometer (Agilent Technologies Deutschland GmbH, Waldbronn, Germany). Namely, a 2.96 mL volume of 0.3 mM ABTS solution in phosphate-citrate buffer (51 mM Na_2_HPO_4_, 24 mM citric acid, pH 5.0) was mixed with a 30 µL volume of 0.1 µM HRP solution in a 3-mL quartz cell of 1 cm pathlength (Agilent Technologies Deutschland GmbH, Waldbronn, Germany). Accordingly, the final concentration of the enzyme in the cell was 1 nM. Then, 8.5 mL of 3% (*w*/*w*) H_2_O_2_ was pipetted into the cell, and spectrum acquisition was started immediately. Absorbance of the solution in the cell was monitored at 405 nm [65]. At this wavelength, the millimolar extinction coefficient of oxidized ABTS amounts to *ε*_405_ = 36.8 mM^−1^ cm^−1^, and its concentration at each time point *t* of the measurement was calculated based on the Beer–Lambert law [66]:

[*oxidized ABTS*] = (*A*_405_(*t*) − *A*_405_(*t =* 0))/(*ε*_405_ × *l*), (2)

where *A*_405_ is the absorbance of the solution in the cell at 405 nm, and *l* is the cell pathlength (*l* = 1 cm).

The behaviour of the HRP enzyme in the ABTS oxidation reaction was estimated on the basis of time dependencies of the concentration of oxidized ABTS, which was calculated based on the absorbance of the solution in the cell at 405 nm (Equation (2)).

## 3. Results

### 3.1. Atomic Force Microscopy

**Figure 3:**
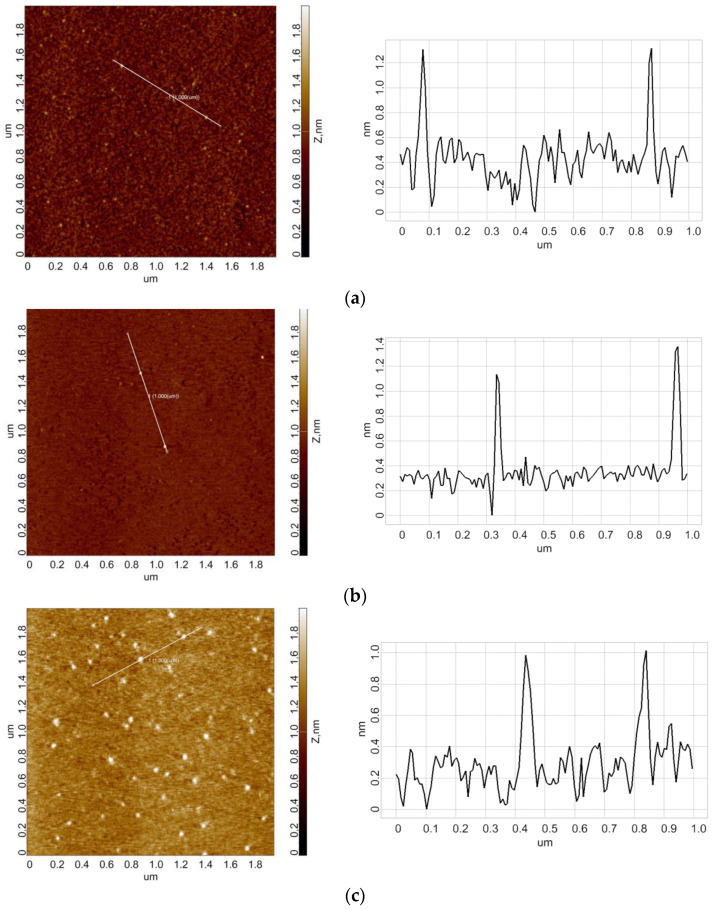
Typical AFM images of mica surface with adsorbed HRP (left) and respective cross-section profiles (right) obtained for HRP solutions incubated either 2 cm above the conical coil (**a**), to the side of the coil (**b**), or 50 m away from the coil ((**c**), control experiment). For all AFM images, the scan size is 2 µm × 2 µm, and the Z scale is from 0 to 2 nm.

Figure 3a,b displays typical AFM images obtained in the experiments with 10^−7^ M HRP solution in 2 mM, pH 7.4 Dulbecco’s modified phosphate buffered saline (PBSD) incubated for three minutes at either 2 cm above the top or at the side of the conical coil with flowing steam. In the control experiments, the HRP solution was incubated 50 m away from the coil (Figure 3c).

**Figure 4:**
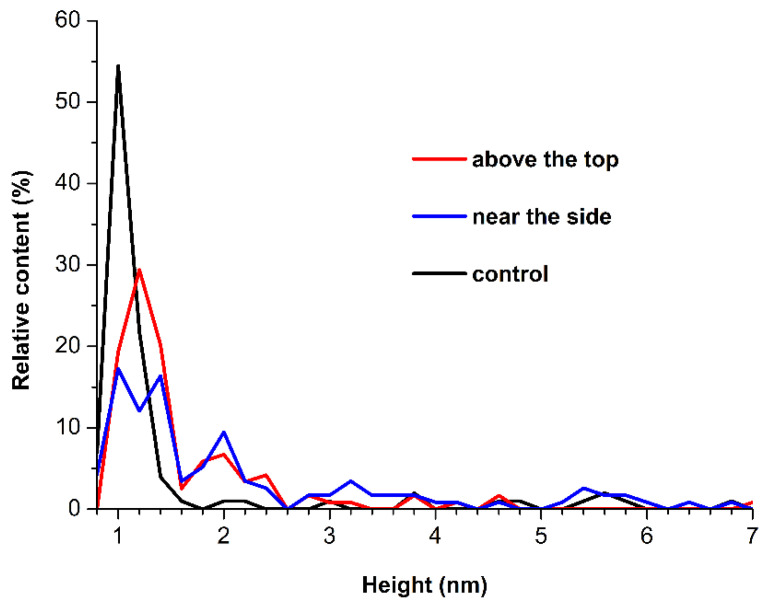
Relative *ρ*(*h*) distributions of the mica-adsorbed HRP particles obtained for the HRP samples incubated either 2 cm above the conical coil (red), to the side of the coil (blue), or 50 m away from the coil (black, control experiment).

The images shown in Figure 3 indicate that in all experiments, HRP adsorbs onto mica in the form of compact objects, whose height typically does not exceed 1.4 nm. After processing the AFM data obtained for all the enzyme samples studied, the respective *ρ*(*h*) distributions were plotted. Figure 4 displays the *ρ*(*h*) distributions obtained for the samples incubated either near or 50 m away from the conical coil.

As can be seen from Figure 4, for the control solution, the majority of objects are 1 nm in height, while the content of objects with heights within the 1.6–2.4 nm range is insignificant. In contrast, for the HRP solution incubated either above the coil or near its side, the respective *ρ*(*h*) curves clearly display a significant increase in the content of higher (1.6 nm to 2.6 nm) objects, which contribute to the right wing of the *ρ*(*h*) distributions. Previously, we showed that in case of direct adsorption of HRP onto mica, objects of 1–1.2 nm height pertain to the monomeric form of HRP, while HRP aggregates on mica are characterized with greater heights [32]. Accordingly, the results of our AFM measurements obtained herein indicate an increased aggregation of HRP on mica after the incubation of its solution near the conical coil with flowing steam.

### 3.2. Spectrophotometry

**Figure 5:**
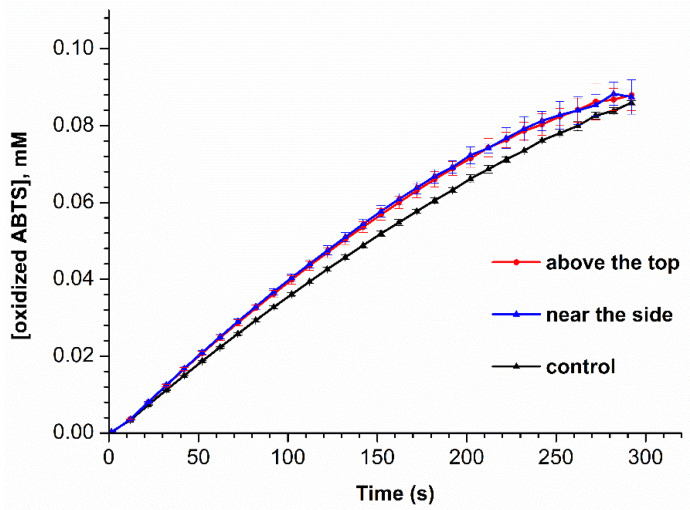
Time dependencies of concentration of oxidized ABTS in the HRP-ABTS-H_2_O_2_ system for HRP samples incubated either 2 cm above the conical coil (red), to the side of the coil (blue), or 50 m away from the coil (black, control experiment). Measurement conditions: HRP:ABTS:H_2_O_2_ = 1 nM:2.5 mM:0.3 mM; pH 5.0; solution absorbance was monitored at 405 nm wavelength; cell pathlength was 1 cm, solution temperature was 25 °C.

HRP activity measurements were performed for all the samples studied by AFM. Figure 5 displays time dependencies of concentration of oxidized ABTS in the HRP:ABTS:H_2_O_2_ system, obtained by measuring the solution absorbance at 405 nm for all the HRP samples studied.

The curves shown in Figure 5 indicate that after five minutes, the absorbance of the HRP:ABTS:H_2_O_2_ reaction mixture is similar for all the enzyme samples studied. Furthermore, it is to be noted that the shape of the curve recorded for the control enzyme sample is slightly different from that of the curves recorded for both the samples incubated in the vicinity of the coil. 

## 4. Discussion

In our present study, the influence of steam flow through a conical coil heat exchanger on the HRP enzyme has been studied. At the coil input, the steam temperature was 100 °C, and after passing the coil top, the temperature dropped down to 70 °C. In our experiments, the samples of HRP solution have been incubated at either 2 cm above the top or 2 cm from the side of the conical coil, while the control sample was incubated 50 m away from the coil. By AFM, an increase in the content of the aggregated form of HRP on mica has been revealed after the incubation of the enzyme near the coil—as compared with the control enzyme sample. Moreover, it is interesting to note that such an incubation has also led to a slight change in the enzyme behaviour in the ABTS oxidation reaction. Namely, the shape of the *A*_405_(*t*) kinetic curve recorded for the control enzyme sample is slightly different from that of the curves recorded for both the samples incubated near the conical coil (either above the coil or near its side). Additionally, the *A*_405_(*t*) curves recorded for both the samples incubated near the coil are barely distinguishable from each other, as their shape is the same. These are the very samples for which a well-pronounced aggregation on mica has been observed by AFM.

These effects can take place at the expense of a change in the degree of enzyme hydration. This phenomenon can be explained in the following way.

The degree of enzyme hydration depends on external conditions. Water is known to be a spin-nonequilibrium mixture of para- and ortho-isomers of H_2_O [67]. It is known to contain ice-like clusters, corresponding to the para-isomers, even at a temperature of about 99 °C [68]. This means that even at high temperatures, water is spin-nonequilibrium. When a heated steam moves through a pipe (which forms a coil), boundary layers form on the inner surface of the pipe. The temperature of the aqueous environment of these layers should change, thus leading to a change in the ratio between ortho- and para-H_2_O isomers. This, in turn, can induce radiation similar to that described in [69]; this happens at the expense of ortho- to para-isomer transitions, which take place owing to quantum-mechanical resonance phenomena. Such a radiation can stimulate enzyme hydration at nearby points, as was noted by Pershin [70,71]. The change in enzyme hydration can also explain the slight change in the behaviour of the enzyme in the ABTS oxidation reaction, since enzyme hydration was reported to be one of the factors influencing enzymatic activity [72,73,74].

The results obtained indicate that steam flow in a conical coil heat exchanger affects the physicochemical properties of HRP enzyme. Since enzymes play key roles in the regulation of processes in human body [44], this phenomenon should be taken into account in the development of equipment involving conical heat exchangers, intended for either research or industrial use with respect to the possible influence on the equipment operators. Moreover, the course of pathological processes is associated with the enzymes participating in the formation of functionally important multiprotein complexes: for instance, inflammatory processes in the body are mediated by the dimeric form of myeloperoxidase [45]. Naturally, if a peroxidase changes its aggregation state under the influence of steam flow in a coiled heat exchanger, then it may influence the course of inflammation-associated pathologies. Furthermore, protein aggregation can lead to changes in hemodynamics in small vessels, but at the same time, it can affect pathological changes associated with the functioning of enzymes in other organs of the body.

## 5. Conclusions

In our AFM experiments reported herein, a 3 min incubation of 0.1 µM aqueous solution of HRP in the vicinity (at a 2 cm distances) of a conical coil heat exchanger, through which a steam flow passed, has been found to cause an increase in the aggregation of individual macromolecules of the enzyme on mica. Moreover, by spectrophotometry, a slight change in the behaviour of the enzyme in the reaction of ABTS oxidation in solution has also been revealed after such an incubation. These effects on the enzyme adsorption and kinetics can be explained by alterations in the enzyme hydration, which were caused by the influence of the electromagnetic field induced triboelectrically by the flow of steam through the heat exchanger. Since conical heat exchangers are known to be used in biosensors and bioreactors (in which enzymes can be utilized), the effects revealed herein should be considered in the development of bioreactors and biosensors (including miniaturized ones) intended for either research or industrial use. 

## Data Availability

Correspondence and requests for materials should be addressed to Y.D.I.
